# Benzene Uptake and *Glutathione S-transferase T1* Status as Determinants of *S*-Phenylmercapturic Acid in Cigarette Smokers in the Multiethnic Cohort

**DOI:** 10.1371/journal.pone.0150641

**Published:** 2016-03-09

**Authors:** Christopher A. Haiman, Yesha M. Patel, Daniel O. Stram, Steven G. Carmella, Menglan Chen, Lynne R. Wilkens, Loic Le Marchand, Stephen S. Hecht

**Affiliations:** 1 Department of Preventive Medicine and Norris Comprehensive Cancer Center, Keck School of Medicine, University of Southern California, Los Angeles, CA, 90032, United States of America; 2 Masonic Cancer Center, University of Minnesota, Minneapolis, MN, 55105, United States of America; 3 Epidemiology Program, Cancer Research Center of Hawai’i, University of Hawai’i, Honolulu, HI, 96813, United States of America; Fudan University, CHINA

## Abstract

Research from the Multiethnic Cohort (MEC) demonstrated that, for the same quantity of cigarette smoking, African Americans and Native Hawaiians have a higher lung cancer risk than Whites, while Latinos and Japanese Americans are less susceptible. We collected urine samples from 2,239 cigarette smokers from five different ethnic groups in the MEC and analyzed each sample for *S*-phenylmercapturic acid (SPMA), a specific biomarker of benzene uptake. African Americans had significantly higher (geometric mean [SE] 3.69 [0.2], p<0.005) SPMA/ml urine than Whites (2.67 [0.13]) while Japanese Americans had significantly lower levels than Whites (1.65 [0.07], p<0.005). SPMA levels in Native Hawaiians and Latinos were not significantly different from those of Whites. We also conducted a genome-wide association study in search of genetic risk factors related to benzene exposure. The *glutathione S-transferase T1* (*GSTT1*) deletion explained between 14.2–31.6% (p = 5.4x10^-157^) and the *GSTM1* deletion explained between 0.2%-2.4% of the variance (p = 1.1x10^-9^) of SPMA levels in these populations. Ethnic differences in levels of SPMA remained strong even after controlling for the effects of these two deletions. These results demonstrate the powerful effect of *GSTT1* status on SPMA levels in urine and show that uptake of benzene in African American, White, and Japanese American cigarette smokers is consistent with their lung cancer risk in the MEC. While benzene is not generally considered a cause of lung cancer, its metabolite SPMA could be a biomarker for other volatile lung carcinogens in cigarette smoke.

## Introduction

Results from the Multiethnic Cohort (MEC) demonstrate that, for the same quantity of cigarettes smoked, particularly at lower levels of smoking, African Americans and Native Hawaiians had a higher risk for lung cancer than Whites while Latinos and Japanese Americans were less susceptible [1]. These variations were evident for all histologic types of lung cancer and in both women and men, but were not seen in non-smokers. Similar results from studies of different designs have been observed previously and the SEER data also demonstrate racial/ethnic differences in lung cancer incidence [2–9]. Multiple studies have examined genetic variation in carcinogen metabolizing genes and DNA repair pathways with respect to lung cancer incidence, with genome-wide association studies highlighting the region of the *CHRNA5*-*CHRNA3*-*CHRNB4* gene cluster on chromosome 15q25 in association with quantity smoked and lung cancer risk [10–15]. These studies, which include ethnic-specific analyses, provide some important mechanistic leads but the degree to which these genetic factors contribute to ethnic differences in smoking-related lung cancer risk has not been established. Our approach uses a combination of carcinogen-specific phenotyping and a genome-wide association study (GWAS) in a subgroup of MEC participants who were cancer-free current smokers. In studies published to date, we have examined urinary biomarkers of uptake of nicotine, a tobacco-specific nitrosamine, acrolein, and crotonaldehyde in these smokers [16–19]. The focus of the study presented here is benzene, a representative volatile carcinogen in cigarette smoke.

Benzene is a human carcinogen and a recognized cause of acute myeloid leukemia and acute non-lymphocytic leukemia [20, 21]. The highest consistent non-occupational exposure to benzene occurs in cigarette smokers; the mainstream smoke of a single cigarette typically contains 15–59 micrograms of benzene [22]. It has been estimated that nearly 90% of benzene exposure in smokers is due to benzene in cigarette smoke [23]. When smokers stopped smoking cigarettes, benzene exposure as measured by urinary *S*-phenylmercapturic acid (SPMA) rapidly decreased by about 80% [24]. There also can be non-occupational exposures to benzene in high traffic areas and near gasoline filling stations, and from contaminated water and food. Occupational exposures to benzene can occur in a variety of settings including the petrochemical industry, around gasoline service stations, and in the rubber and paint industries, but these are relatively rare in the U.S. [20, 21, 23, 25]. Although benzene is not generally considered to play a major role in lung cancer etiology in smokers, compared for example to tobacco-specific nitrosamines and polycyclic aromatic hydrocarbons [26], it could be a biomarker for other volatile constituents of cigarette smoke involved in lung cancer etiology.

**Fig 1:**
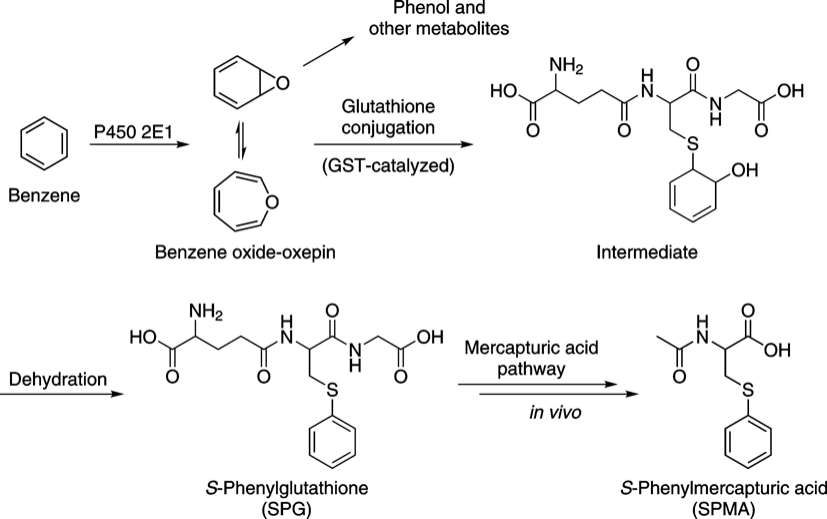
Overview of benzene metabolism to SPMA.

Benzene requires metabolic activation to exert its carcinogenic effects (Fig 1). The critical and requisite intermediate benzene oxide can be detoxified by reaction with glutathione, catalyzed by glutathione *S*-transferases (GSTs), such as *GSTT1* and *GSTM1*. The glutathione conjugate of benzene oxide is processed by a series of enzymes resulting in the excretion of SPMA in urine. SPMA is an accepted and specific biomarker of benzene exposure [20, 27, 28].

Polymorphisms exist in *GST*s, including deletion of the *GSTT1* and *GSTM1* genes, leading to the logical hypothesis that there could be corresponding effects on levels of SPMA in urine (reviewed in [29, 30]). With respect to *GSTT1* polymorphisms and SPMA levels in urine, ten previous studies ranging in size from 37 to 386 subjects have reported an effect of *GSTT1* null status on urinary SPMA levels. Consistently, higher levels of SPMA were found in the urine of subjects exposed occupationally or environmentally to benzene and who were *GSTT1* positive compared to those with the null genotype [31–40]. Smaller effects on SPMA levels were observed in studies of individuals with the *GSTM1* deletion [29].

In the study reported here of 2,239 cigarette smokers from the five ethnic groups of the MEC, we assessed benzene uptake by quantifying SPMA in urine. In tandem with these SPMA analyses, we carried out a GWAS exploring the effects of common genetic variants on urinary SPMA levels. This study is by far the largest yet reported to investigate SPMA levels in genotyped subjects, allowing us to confidently analyze ethnic differences in benzene uptake, as indicated by urinary SPMA, and determine the effects of genotype on SPMA levels. The results demonstrate both the power and the limitations of the SPMA biomarker while also providing new data on benzene uptake in smokers from populations with differing risks for lung cancer.

## Materials and Methods

The Institutional Review Boards at the University of Southern California, the University of Hawaii, and the University of Minnesota approved of the study protocol. The participants provided written consent to participate in the study. The Institutional Review Boards at the University of Southern California and at the University of Hawaii approved of the consent procedure.

### Study Population

The study subjects are MEC participants who were current smokers at the time of biospecimen collection. The MEC is a prospective cohort study established to investigate the association of lifestyle and genetic factors with chronic diseases [41] and is comprised of 215,251 men and women between the ages of 45 to 75 at baseline, primarily belonging to five ethnic/racial groups: African Americans, Native Hawaiians, Whites, Latinos, and Japanese Americans. Between 1993 and 1996, potential participants were identified in Hawaii and California (primarily Los Angeles County) through drivers’ license files, voter registration lists, and Health Care Financing Administration files. Each participant completed a mailed, self-administered questionnaire regarding demographic, dietary, lifestyle, and other exposure factors.

This specific study comprises a subgroup of the MEC participants who were cancer-free current smokers at the time of urine collection. Approximately 10 years after cohort entry, 2,393 current smokers with no cancer diagnosis participated in the MEC bio-specimen sub-cohort by providing a blood sample and overnight (subjects recruited in Hawaii—mostly Whites, Native Hawaiians and Japanese Americans) or first morning urine (subjects recruited in California–mostly African Americans and Latinos) and completing an epidemiologic questionnaire that included a history of daily cigarette smoking during the past two weeks, smoking duration, and a record of current medications. The overnight urine collection started between 5–9 pm (depending on the subject) and included all urine passed during the night as well as the first morning urine. All urine was kept on ice until processing. Aliquots were subsequently stored in a -80°C freezer until analysis.

### Phenotype Measurements

Analysis of SPMA in urine was performed by liquid chromatography-tandem mass spectrometry essentially as described [42], with the following modifications: 1. [D_5_]SPMA (12.5 ng, Toronto Research Chemicals) was added to the urine samples as internal standard; 2. Following washing of the 96-well Oasis MAX plates with 0.7 ml of 30% methanol in 2% aqueous formic acid to elute 3-hydroxypropylmercapturic acid (3-HPMA) and 3-hydroxy-1-methylpropylmercapturic acid (HMPMA), the plates were washed with 0.7 ml 50% methanol in 2% aqueous formic acid and this wash was discarded. The plates were then washed with 0.7 ml of 90% methanol in 2% formic acid to collect the fraction containing SPMA and the internal standard; 3. The MS transitions monitored were *m/z* 238.05 → *m/z* 109.05 for SPMA and *m/z* 243.05 → *m/z* 114.05 for [D_5_]SPMA. The limit of quantitation was 0.1 pmol/ml.

Methods of analysis for total nicotine equivalents (TNE, the sum of nicotine, cotinine, 3′-hydroxycotinine and their glucuronides, and nicotine *N*-oxide), 4-(methylnitrosamino)-1-(3-pyridyl)-1-butanol (total NNAL, a biomarker of uptake of the tobacco-specific carcinogen NNK), 3-HPMA, a biomarker of uptake of the volatile toxicant acrolein, and HMPMA, a biomarker of uptake of the volatile carcinogen and toxicant crotonaldehyde, in the urine of MEC subjects have been described [16, 42–44].

Individuals who smoked only to a limited degree (determined by TNE less than 1.4 nmol/ml, n = 80) were excluded from the study. Those with SPMA below the limit of detection (0.1 pmol/ml) were also excluded (n = 139).

### Genotyping and Quality Control

A total of 2,418 current smokers were genotyped using the Illumina Human1M-Duo BeadChip (1,199,187 SNPs), as previously described [18]. The genotyping quality control consisted of 1) removing individual samples with ≥2% of genotypes not called (n = 8), 2) removing SNPs ≤98% call rate (n = 67,761), 3) removing known duplicate samples (n = 25), 4) excluding samples with close relatives (as determined by estimated IBD status in pair wise comparisons of samples; n = 59), and samples with conflicting or indeterminate sex (n = 7). Genotyping of the *GSTT1* and *GSTM1* deletions was performed by TaqMan and run on the 7900HT Fast Real-Time System (Life Technologies, Foster City, CA). Copy number counts were calculated using Life Technologies CopyCaller v2.0 software. Approximately 5% of blind duplicates were included for quality control. Genotyping of the *GSTT1* and *GSTM1* deletion polymorphisms was successful in 2,111, and 2,225 individuals, respectively. Test for Hardy Weinberg Equilibrium was met for all five populations for *GSTM1* (p>0.05), Latinos did not meet these criteria for *GSTT1* (p = 0.01).

### Imputation to Estimate Unmeasured Genotypes

Imputation was performed using SHAPEIT [45] and IMPUTE2 [46] to a cosmopolitan reference panel from the 1000 Genomes Project (1KGP; March, 2012). We included SNPs with an IMPUTE2 info score of ≥0.30 and minor allele frequency (MAF) >1% in any MEC ethnic group. A total of 11,892,802 SNPs/indels with a frequency >1% in any single ethnic population (1,131,426 genotyped and 10,761,376 imputed) were included in the analysis.

### Statistical Analysis

Least-square means (or geometric means) were estimated and compared between populations for the smoking variables. Principal components were estimated using 19,059 randomly selected autosomal SNPs with frequency ≥ 2% in the combined multiethnic sample [47]. The 10 leading eigenvectors from this matrix were included in the analysis to adjust for population stratification. The per allele association of each SNP/indel with geometric mean SPMA levels was evaluated using linear regression models, with adjustment for age at the time of urine collection, sex, reported ethnicity, TNE, BMI, and the first 10 principal components described above. A p-value cut-off of 5x10^-8^ was used to establish genome-wide significance. Conditional models were used for regions with multiple associated variants (at p<5x10^-8^). Ethnic-specific analyses were performed to search for loci that may be important in individual populations and tests of heterogeneity by ethnic group were performed by including an interaction term between ethnicity and variant in regression models. We also conducted analyses among subjects homozygous for the *GSTT1* and *GSTM1* non-null alleles to examine associations with variants located in the deleted region. We also report on the associations with variants in candidate gene regions known to be involved in benzene metabolism (e.g., *CYP2E1*). R^2^ value was used to assess the percentage of variation of SPMA accounted for by the variants examined. To examine correlations between SPMA and other biomarkers, Pearson’s partial correlations (r) were reported and adjusted for age, gender, BMI and race. Genomic control [48] (estimation of over-dispersion parameter λ) was used to assess adequacy of control for population stratification and other aspects of the behavior of tests SNP effects in the GWAS data.

## Results

A total of 2,239 smokers (364 African Americans, 311 Native Hawaiians, 437 Whites, 453 Latinos, and 674 Japanese Americans) were included in the main analysis (Table 1). Significant differences in smoking, as expressed in cigarettes per day, among the ethnic groups in this sample have been reported previously [16–18]. Among both men and women, Whites reported smoking the highest number of cigarettes per day followed by Native Hawaiians, Japanese Americans, African Americans and Latinos (Table 1).

**Table 1:** Descriptive characteristics of the multiethnic sample of smokers.

n = 2,239		African Americans		Native Hawaiians		Whites		Latinos		Japanese Americans	
		n	Mean [SE]	n	Mean [SE]	n	Mean [SE]	n	Mean [SE]	n	Mean [SE]
Age (yrs)	*All*	364	64.86 [0.38] *	311	61.36 [0.41] ***	437	63.69 [0.35]	453	65.53 [0.34] ***	674	63.60 [0.29]
	*Male*	111	63.49 [0.65]	114	63.08 [0.64]	190	63.36 [0.50]	237	66.43 [0.45]	388	63.62 [0.36]
	*Female*	253	65.39 [0.47]	197	60.28 [0.54]	247	63.92 [0.48]	216	64.58 [0.51]	286	63.69 [0.47]
CPD^a^	*All*	364	11.72 [0.48] ***	311	15.52 [0.52] ***	437	18.02 [0.43]	453	9.31 [0.43] ***	674	13.76 [0.37] ***
	*Male*	111	11.98 [0.91] ***	114	17.00 [0.91] **	190	21.00 [0.70]	237	10.87 [0.64] ***	388	15.58 [0.51] ***
	*Female*	253	10.79 [0.53] ***	197	14.27 [0.61]	247	15.47 [0.53]	216	8.14 [0.57] ***	286	12.05 [0.53] ***
TNE^b^	*All*	364	44.94 [2.00] ***	311	30.61 [1.48]	437	34.44 [1.38]	453	31.28 [1.25]	674	25.08 [0.86] ***
	*Male*	111	47.97 [3.85]	114	33.04 [2.65] *	190	42.55 [2.64]	237	33.76 [1.91] *	388	27.99 [1.25] ***
	*Female*	253	41.20 [2.16] ***	197	28.86 [1.71]	247	28.90 [1.51]	216	29.65 [1.67]	286	22.76 [1.20] **
Creatinine^c^	*All*	364	90.18 [2.87]***	311	59.08 [2.03]	437	57.66 [1.66]	453	70.63 [2.01]***	674	52.66 [1.24]*
	*Male*	111	110.4 [6.44]***	114	72.88 [4.22]	190	71.85 [3.26]	237	84.24 [3.43]**	388	65.66 [2.07]
	*Female*	253	75.46 [2.81]***	197	49.37 [2.08]	247	47.70 [1.78]	216	61.03 [2.44]***	286	43.18 [1.53]
SPMA/ml^d^	*All*	354	3.69 [0.2] ***	296	2.42 [0.14]	418	2.67 [0.13]	431	2.86 [0.14]	601	1.65 [0.07] ***
	*Male*	109	4.52 [0.44]**	110	2.42 [0.24]	182	3.09 [0.24]	225	2.78 [0.19]	355	1.73 [0.09] ***
	*Female*	245	3.34 [0.22]**	186	2.33 [0.17]	236	2.42 [0.16]	206	2.93 [0.20] *	246	1.60 [0.10] ***
SPMA/mg^e^	*All*	354	4.06 [0.19]	296	4.06 [0.21]	418	4.56 [0.19]	431	3.96 [0.17] *	601	3.05 [0.11] ***
	*Male*	109	4.10 [0.34]	110	3.32 [0.28] *	182	4.16 [0.28]	225	3.20 [0.19] **	355	2.56 [0.12] ***
	*Female*	245	4.38 [0.24]	186	4.68 [0.29]	236	5.05 [0.28]	206	4.75 [0.28]	246	3.61 [0.20] ***

^a^ CPD = cigarettes/day; least square means for CPD have been adjusted for BMI, age (and gender where appropriate).

^b^ TNE (total nicotine equivalents) is the sum of total nicotine, total cotinine, total 3′-hydroxycotinine, and nicotine *N*-oxide expressed as nmol/ml; geometric least square means for TNE have been adjusted for BMI, age (and gender where appropriate).

^c^ Creatinine expressed as mg/dl; geometric least square means of creatinine have been adjusted for BMI, age, TNE (and gender where appropriate).

^d^ SPMA (*S*-Phenyl mercapturic Acid) expressed as pmol/ml; geometric least square means of SPMA have been adjusted for BMI, age, TNE (and gender where appropriate).

^e^ SPMA expressed as pmol/mg creatinine; geometric least square means of SPMA have been adjusted for BMI, age, TNE (and gender where appropriate).

* P-values across ethnic groups (with Whites as the reference) were indicated where significant as *p < 0.05

**p<0.005 and

***p<0.0005.

As also reported previously, levels of TNE were highest among the African Americans and lowest in the Japanese Americans compared to the Whites (Table 1) [16, 18]. We also noted significantly higher levels of creatinine in African Americans (p = 1x10^-24^) and Latinos (p = 8x10^-7^) compared to Whites with significantly lower levels observed in Japanese (p = 0.015) (Table 1). For simplicity, because of this large variability in creatinine levels across populations, SPMA was expressed per ml of urine rather than per mg of creatinine.

SPMA was significantly correlated with several other urinary biomarkers in the MEC subjects: TNE, total NNAL, 3-HPMA, and HMPMA (Table 2). The strongest correlations were with TNE and total NNAL, but all were highly significant.

**Table 2:** Partial Correlations Among SPMA and Other Biomarkers

	N	SPMA & TNE^a^	N	SPMA & Total NNAL^a^	N	SPMA & 3-HPMA^a^	N	SPMA & HMPMA^a^
Overall	2100	0.57	2048	0.47	2070	0.26	2070	0.28
African Americans	354	0.61	343	0.47	346	0.34	346	0.34
Native Hawaiians	296	0.52	290	0.39	294	0.26	294	0.31
European Americans	419	0.63	408	0.54	410	0.21	410	0.26
Latinos	431	0.66	418	0.57	422	0.30	422	0.29
Japanese Americans	601	0.42	589	0.36	598	0.19	598	0.23
GSTT1 = 0	524	0.53	515	0.49	523	0.22	523	0.24
GSTT1 = 1	937	0.71	912	0.6	919	0.31	919	0.36
GSTT1 = 2	514	0.74	498	0.62	504	0.31	504	0.35
GSTM1 = 0	1005	0.54	973	0.48	992	0.18	992	0.25
GSTM1 = 1	861	0.63	847	0.54	850	0.28	850	0.29
GSTM1 = 2	221	0.63	215	0.49	215	0.24	215	0.27

^a^ All partial correlations are significant at p < 0.0001 and have been adjusted for age, gender, BMI (and race where appropriate).

We observed large differences in mean SPMA levels per ml urine across populations, even after adjusting for TNE, with African Americans having 38% higher levels (p = 1.4x10^-5^) and Japanese Americans having 38% lower levels (p = 2x10^-13^) than Whites. Similar results were obtained when the data were expressed as median SPMA levels. Given the variability in creatinine, lesser differences in SPMA were observed when adjusted for creatinine levels (Table 1). Still however, levels in Japanese remained significantly lower than levels in Whites.

In the GWAS analysis of SPMA we observed little evidence of inflation in the test statistic in the overall multiethnic sample (λ = 1.0; S1 Fig) or in any single ethnic group (0.97 ≤ λ’s ≤ 1.0). We detected associations at p<5x10^-8^ with 403 variants located between 24.2–24.4 Mb near the *GSTT1* gene on chromosome 22q11 (S2 Fig) and 1 variant near the *GSTM1* gene at 1p13 (S3 Fig; S1 Table). The highly significant association observed at 22q11 was explained by the *GSTT1* deletion (n = 1,975, beta per allele = 2.06 pmol/ml, p = 6.0x10^-107^; Table 3). The r^2^ between the deletion and the associated variants (P<5x10^-8^) ranged from 0.02 to 0.43 in the multiethnic sample and no secondary signals (at p<1x10^-3^) were detected after conditioning on the deletion in the multiethnic sample or in any ethnic group (S4 Fig). The deletion allele, which is associated with lower SPMA levels, varies in frequency across populations from 0.40 in Latinos to 0.66 in Japanese (Table 3). We also did not detect any highly significant associations (p<1.7x10^-7^) with SNPs or indels among those without the *GSTT1* deletion (n = 514 homozygotes) which suggests that alternate forms of functional variation in the region are likely to have only a minor impact on the regulation or activity of *GSTT1*. We also performed the analysis of the genetic data with SPMA levels expressed as pmol/mg creatinine; the results vary only marginally (S2 Table).

**Table 3:** SPMA levels by GST deletion genotype.

	**SPMA (pmol/mL)**												
***GSTT1 deletion***	**African Americans**		**Native Hawaiians**		**Whites **		**Latinos**		**Japanese Americans**		**Total**		**P-het**^b^
	**n**	**Mean**^a^	**n**	**Mean**^a^	**n**	**Mean**^a^	**n**	**Mean**^a^	**n**	**Mean**^a^	**n**	**Mean**^a^	** **
**All**	329	3.71	265	2.43	404	2.69	415	2.88	562	1.66	1975		** **
**0**	68	1.56	69	1.03	68	1.09	73	1.04	246	0.9	524	1.1	
**1**	174	4.10	130	3.07	198	2.47	182	2.65	253	2.33	937	2.87	
**2**	87	5.89	66	4.01	138	4.59	160	4.79	63	3.53	514	4.56	
**Null Frequency**	0.47		0.51		0.41		0.40		0.66				
**Beta per allele**	1.84		1.97		2.07		1.98		2.18		2.06		
**P-trend**^b^	8.0x10^-14^		4.9x10^-18^		8.8x10^-25^		2.8x10^-23^		1.2x10^-36^		6.0x10^-107^		0.328
**Variation Explained**	14.24%		31.56%		23.87%		20.45%		27.18%		20.88%		
	**SPMA (pmol/mL)**												
***GSTM1* deletion**	**African Americans**		**Native Hawaiians**		**Whites**		**Latinos**		**Japanese Americans**		**Total**		**P-het**^b^
	**n**	**Mean**^a^	**n**	**Mean**^a^	**n**	**Mean**^a^	**n**	**Mean**^a^	**n**	**Mean**^a^	**n**	**Mean**^a^	
**All**	353	3.73	290	2.46	417	2.69	430	2.88	597	1.66	2087		
**0**	103	3.2	185	2.15	220	2.33	196	2.58	301	1.41	1005	2.23	
**1**	168	3.84	91	3.2	158	3.10	192	3.06	252	1.97	861	2.95	
**2**	82	4.2	14	2.66	39	3.35	42	3.77	44	1.86	221	3.14	
**Null Frequency**	0.53		0.79		0.72		0.68		0.72				
**Beta per allele**	1.13		1.29		1.28		1.2		1.24		1.2		
**P-trend**^b^	0.13		9.2x10^-3^		1.4x10^-3^		0.02		1.6x10^-3^		3.3x10^-9^		0.896
**Variation Explained**	0.20%		1.44%		1.34%		1.46%		2.40%		1.26%		

^a^ All Means are expressed as geometric least squares means of SPMA adjusted for age, gender, total nicotine equivalents, BMI and race (where appropriate).

^b^ P-values for trend and heterogeneity have been adjusted for age, gender, total nicotine equivalents, BMI, PCs and race (where appropriate).

The second region of association was at 1p13 where only a single imputed variant (indel at position 110223001 bp; info score = 0.8) was found to be associated with SPMA at p<5x10^-8^ in the multiethnic sample (frequency range 0.27–0.47 across populations, beta = 0.81 pmol/mL per allele, p = 1.48x10^-10^; S3 Fig). This variant was correlated with the *GSTM1* deletion polymorphism (r^2^ of 0.58 in the multiethnic sample), which was similarly associated with lower SPMA levels (n = 2,087, beta per allele = 1.20 pmol/mL, p = 3.3x10^-9^; Table 3). The indel was no longer significantly associated with SPMA after conditioning on the large deletion polymorphism (S5 Fig). The deletion allele, which is associated with lower SPMA levels, varies in frequency across populations from 0.53 in African Americans to 0.79 in Native Hawaiians. As with 22q11 and the *GSTT1* deletion, we did not detect any significant associations (p<3.4x10^-7^) with SNPs or indels among those without the *GSTM1* deletion (n = 221 homozygotes).

In ethnic-specific analyses, a cluster of ten highly correlated variants (r^2^ ≥0.8) were significant at p<5x10^-8^ within *POU4F1-AS1* at chromosome 13q31 in Latinos. All variants were imputed (imputation quality, info scores ≥ 0.94) and are common in all five populations (freq>0.7); however, these SNPs were only associated with SPMA levels in Latinos (beta>0.62, p ≥ 1.85x10^-8^; beta>1.01 and p-value>0.23 in all other populations).

Given the importance of CYP2E1 in benzene metabolism and the previously reported associations with polymorphisms in *CYP2E1* and benzene metabolite levels we also examined variation at this locus. We observed little evidence of an association with common alleles in this region (within 200 kb of *CYP2E1*). Through a literature review, we created a composite list of 13 SNPs reported to be associated with benzene metabolism; none of these SNPs were found to be associated with SPMA (p< 0.05; S3 Table). The results were similar among those with or without the *GSTT1* deletion polymorphism (data not shown).

Combined, the baseline covariates age, sex, BMI, TNE, cigarettes per day, ethnicity and the first 10 principal components explained 37% of variability in SPMA. Ethnicity and principal components accounted for ~6% of the variability (Table 4). When adjusted for these baseline covariates, cigarettes per day and TNE were both highly associated with SPMA (p = 2.0x10^-14^ and p = 2.1x10^-176^, respectively), though cigarettes per day only explains ~2.5% of the variability in SPMA, whereas TNE explains 29.4%. In the multivariate model, the *GSTT1* deletion accounted for an additional 20.9% of the variability in levels of SPMA in smokers, with the proportion explained ranging from 14.2% in African Americans to 31.6% in Native Hawaiians (p_het_ = 0.33; Table 3). Although genome-wide significant, the contribution of the *GSTM1* deletion was more modest, and could explain only 1.3% of the variation in the multiethnic sample (range across populations: 0.2–2.4%). Together, the *GSTT1* and *GSTM1* deletion polymorphisms explain ~22% of the variation in SPMA levels in this multiethnic sample of smokers, which ranges across ethnic groups from 14.4% in African Americans to 33.0% in Native Hawaiians (Table 3).

**Table 4:** Geometric least square means of SPMA by population and percent variation explained by smoking and GST genotypes.

SPMA (pmol/mL)												
Model		Overall Percent Variation Explained	African Americans		Native Hawaiians		Whites		Latinos		Japanese Americans	
			n	Mean^a^	n	Mean^a^	n	Mean^a^	n	Mean^a^	n	Mean^a^
Sex + Age + BMI + TNE^b^ + CPD^b^		37.21%	329	3.9	264	2.42	403	2.6	414	2.86	562	1.6
	P-values		4.2x10^-7^	4.2x10^-7^	0.38	0.38	-	-	0.26	0.26	6.3x10^-13^	6.3x10^-13^
+ *GSTT1* deletion		20.87%	329	3.62	264	2.42	403	2.28	414	2.47	562	2.02
	P-values		5.7x10^-12^	5.7x10^-12^	0.39	0.39	-	-	0.22	0.22	0.05	0.05
+ *GSTM1* deletion		1.40%	329	3.38	264	2.52	403	2.31	414	2.46	562	2.05
	P-values		1.1x10^-8^	1.1x10^-8^	0.21	0.21	-	-	0.32	0.32	0.04	0.04

^a^ All Means are expressed as geometric least squares means of SPMA adjusted for age, gender, BMI, TNE & CPD.

^b^ TNE,- total nicotine equivalents; CPD, cigarettes per day.

**Fig 2:**
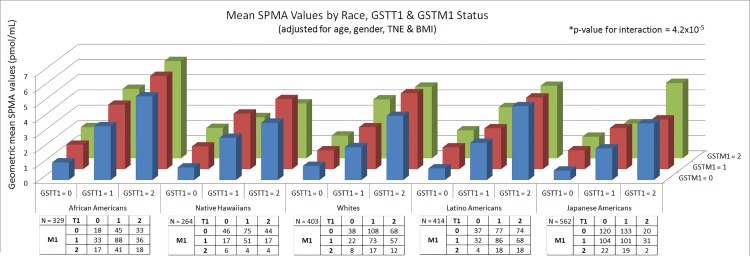
*GSTT1* and *GSTM1* genotype and SPMA levels in each population.

In examining the combined effects of the *GSTT1* and *GSTM1* deletions, SPMA values were lowest among Japanese Americans with null genotypes for both deletions (0.62 pmol/mL) and highest amongst African Americans who were wild-type (6.4 pmol/mL), a 10-fold difference (Fig 2). We observed modest evidence of a statistical interaction between the *GSTT1* and *GSTM1* deletions with SPMA levels (p = 1.2x10^-5^), though the interaction only explained 0.86% of the variability in SPMA. Overall, ~ 60% of the variability in SPMA could be accounted for by the covariates and both deletion polymorphisms.

Despite the highly significant association between the *GSTT1* deletion polymorphism (and to a lesser degree the *GSTM1* polymorphism) and SPMA levels, and the variability in the prevalence of these polymorphisms across populations, they could not account for the large ethnic differences in SPMA levels (Table 4, Fig 2). Compared to Whites, SPMA levels in African Americans remained higher (p<0.0001) while levels in Japanese remained lower (p = 0.04), with the magnitude of these differences not being substantially altered when adjusting for the *GST* deletions. SPMA levels in Latinos and Native Hawaiians were similar to those of Whites.

## Discussion

As in our studies demonstrating statistically higher levels of TNE and total NNAL in African Americans and lower levels in Japanese Americans than in Whites [16, 17], we report here that levels of SPMA, an established and specific biomarker of uptake of the volatile human carcinogen benzene, are significantly higher in African American smokers than in White smokers and significantly lower in Japanese American smokers than in White smokers, even after correction for the effects of variants in the *GSTT1* and *GSTM1* genes. While benzene is well established as a cause of leukemia, it is not generally considered a cause of lung cancer in humans. However, it does cause lung tumors (as well as tumors at other sites) in mice, and some studies indicate its possible involvement in human lung cancer etiology [20]. Perhaps more importantly, benzene uptake as indicated by urinary SPMA, could be a biomarker for other volatile carcinogens in cigarette smoke, such as 1,3-butadiene, which causes lung tumors in mice and has been evaluated as an important carcinogen in cigarette smoke [49–51]. Thus, a single analysis of SPMA could potentially replace multiple analyses of other volatile carcinogen metabolite biomarkers.

The U.S. National Toxicology Program conducted two year carcinogenesis studies of benzene in F-344 rats and B6C3F1 mice. The doses used were 0, 25, 50, or 100 mg/kg body weight of benzene, administered by gavage in corn oil 5 days per week for 103 weeks. Significant incidences of tumors compared to vehicle controls were observed at multiple sites including the hematopoietic system in both rats and mice. Among these, lung tumors were observed only in mice. Significantly increased incidences of alveolar/bronchiolar carcinomas and adenomas were reported, mainly in the mice treated with the highest dose [52]. While statistically significant, the carcinogenic effect of benzene to the rodent lung is far weaker than that of NNK or NNAL [53].

A major finding of the GWAS presented here was the highly significant association of the *GSTT1* deletion on chromosome 22q11 and SPMA levels, which explained up to 31.6% of the variation in SPMA levels, depending on the ethnic group. SPMA is a specific biomarker of benzene uptake, formed by glutathione detoxification of the requisite intermediate benzene oxide, followed by normal metabolic processing of the glutathione conjugate (Fig 1). While this effect of genotype has been noted before, our study is the largest and most definitive [31–40]. The stronger effect of *GSTT1* than *GSTM1* deletion observed here is consistent with our metabolic studies which demonstrate that GSTT1 is a better catalyst of benzene oxide conjugation than GSTM1 [54]. The size of our study allowed us to analyze ethnic differences in SPMA levels correcting for each genotype. As summarized in Table 4 and Fig 2, even after this correction, SPMA levels were significantly higher in African-Americans than in Whites and significantly lower in Japanese Americans than in Whites.

Ethnic differences in *GSTT1* have been observed previously [55]. The prevalence of the null genotype was 64.4% in Chinese, 60.2% in Koreans, 21.8% in African Americans, 20.4% in Caucasians, and 9.7% in Mexican Americans. These results are generally consistent with ours (Table 3) in which the highest null frequency was observed in Japanese Americans (66%) and Native Hawaiians (51%).

The strong effect of *GSTT1* genotype on SPMA levels presents a potential problem in smaller studies interpreting this biomarker as related to benzene uptake. *GSTT1* catalysis of the reaction between benzene oxide and glutathione is a detoxification mechanism for benzene, as benzene oxide is widely recognized as a significant and critical intermediate in benzene carcinogenesis [56, 57]. SPMA levels are affected both by benzene exposure and *GSTT1* genotype. Higher benzene exposure leads to higher levels of urinary SPMA, but *GSTT1* null status, which should *increase* risk for benzene induced toxicity and carcinogenicity (because more benzene oxide will be available to express its deleterious cellular effects) will *decrease* levels of urinary SPMA, as clearly seen in this study. This conundrum could be a problem in smaller studies or those in which genotyping information is not available. An alternate measure of benzene exposure is urinary benzene, which compares well to SPMA in specificity to benzene exposure, but is more difficult to quantify because of its volatility [30].

In this multiethnic sample, which is modest in size for a GWAS, we had limited statistical power to detect a genetic factor that accounts for a small fraction of the variation (R^2^) in SPMA levels. For example, in the entire sample of 2,239 smokers, we had 80% power to detect an R^2^ of 1.8%, at p<5x10^-8^ (allowing for multiple comparisons). The ethnic group specific sample sizes ranged from 311–674 participants so that detectable R^2^ values in any one ethnic group ranged from 6% to 11%. Revealing additional common variants that convey modest effects or less common alleles <5% that may be ethnic specific and which may contribute to population differences in SPMA levels will require substantially larger studies in these racial/ethnic populations.

There were some other limitations to our study. Slightly different urine collection methods–overnight for most of the Native Hawaiians, Whites and Japanese Americans versus first morning for the other two groups–were used. It is possible that these differences might have affected the levels of SPMA in these two groups relative to the others. However, SPMA values did not differ according to collection method when comparing 96 Japanese collected in Los Angeles using first morning urines to the Japanese samples (N = 578) from Hawaii measured from overnight urine collection. In addition, levels of SPMA strongly correlated with those of 3-HPMA and HMPMA (Table 2), yet 3-HPMA and HMPMA were as high in Native Hawaiians as in African Americans [19], while SPMA was significantly lower in Native Hawaiians than in African Americans. Another limitation relates to expressing the results per ml urine rather than per mg creatinine. This mode of expression, which can introduce unwanted variability related to extent of hydration, was necessary because of the wide differences in creatinine seen among some of the ethnic groups collected in the same location (e.g., between African Americans and Latinos collected in Los Angeles, or between Whites and Japanese Americans, collected primarily in Hawaii) which could not be explained by differences in collection method. Twenty-four hour urine samples would have been the preferable method of comparing SPMA levels, but these were not available.

In summary, the results of this study demonstrate that uptake in smokers of the volatile cigarette smoke constituent benzene, as measured by the specific biomarker SPMA, is highest in African Americans, intermediate in Whites, and lowest in Japanese Americans, consistent with their previously determined levels of TNE and total NNAL. Our GWAS convincingly demonstrated the strong effect of *GSTT1* genotype on urinary levels of SPMA, but this did not affect our conclusion regarding ethnic differences in benzene uptake among the MEC smokers.

## Supporting Information

S1 Fig Quantile-Quantile plot of observed and expected–log_10_ transformed p-values from association between SPMA levels and genotyped or imputed alleles from the multiethnic GWAS analysis. (TIF)

S2 Fig Plot of *GSTT1* in our multi-ethnic sample with European LD values. (TIF)

S3 Fig Plot of *GSTM1* in our multi-ethnic sample with European LD values. (TIF)

S4 Fig Plot of Chromosome 22 results, adjusted for *GSTT1* deletion, with European LD values. (TIF)

S5 Fig Plot of 1q13, adjusted for *GSTM1* deletion, with African LD values. (TIF)

S1 Table List of 404 globally significant associations (p < 5E-8) for S-Phenyl Mercapturic Acid. (CSV)

S2 Table SPMA levels by GST deletion genotype. (XLSX)

S3 Table List of overall associations for 13 reported CYP2E1 SNPs. (CSV)
