# Supplementary figures and images for: Benzene Uptake and Glutathione S-transferase T1 Status as Determinants of S-Phenylmercapturic Acid in Cigarette Smokers in the Multiethnic Cohort
Source: PLoS One. 2016 Mar 9;11(3):e0150641. doi: 10.1371/journal.pone.0150641 (PMC4784986; doi:10.1371/journal.pone.0150641)

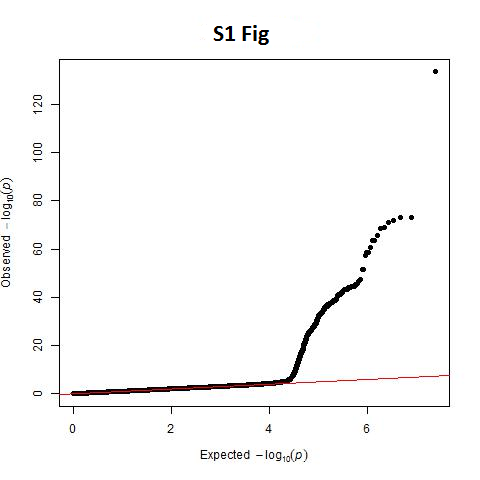

Supplement: S1 Fig — (TIF) [file pone.0150641.s001.tif]

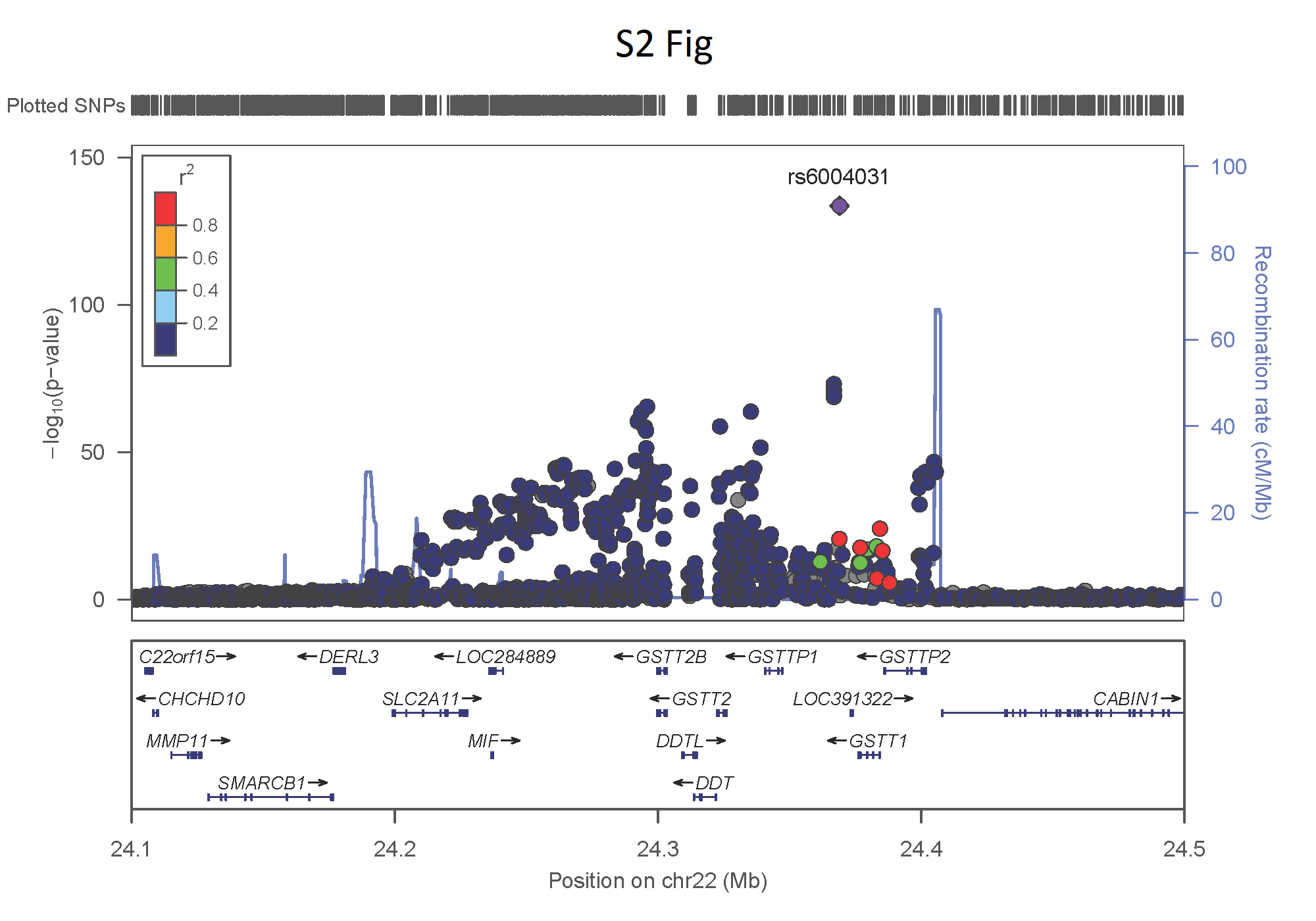

Supplement: S2 Fig — (TIF) [file pone.0150641.s002.tif]

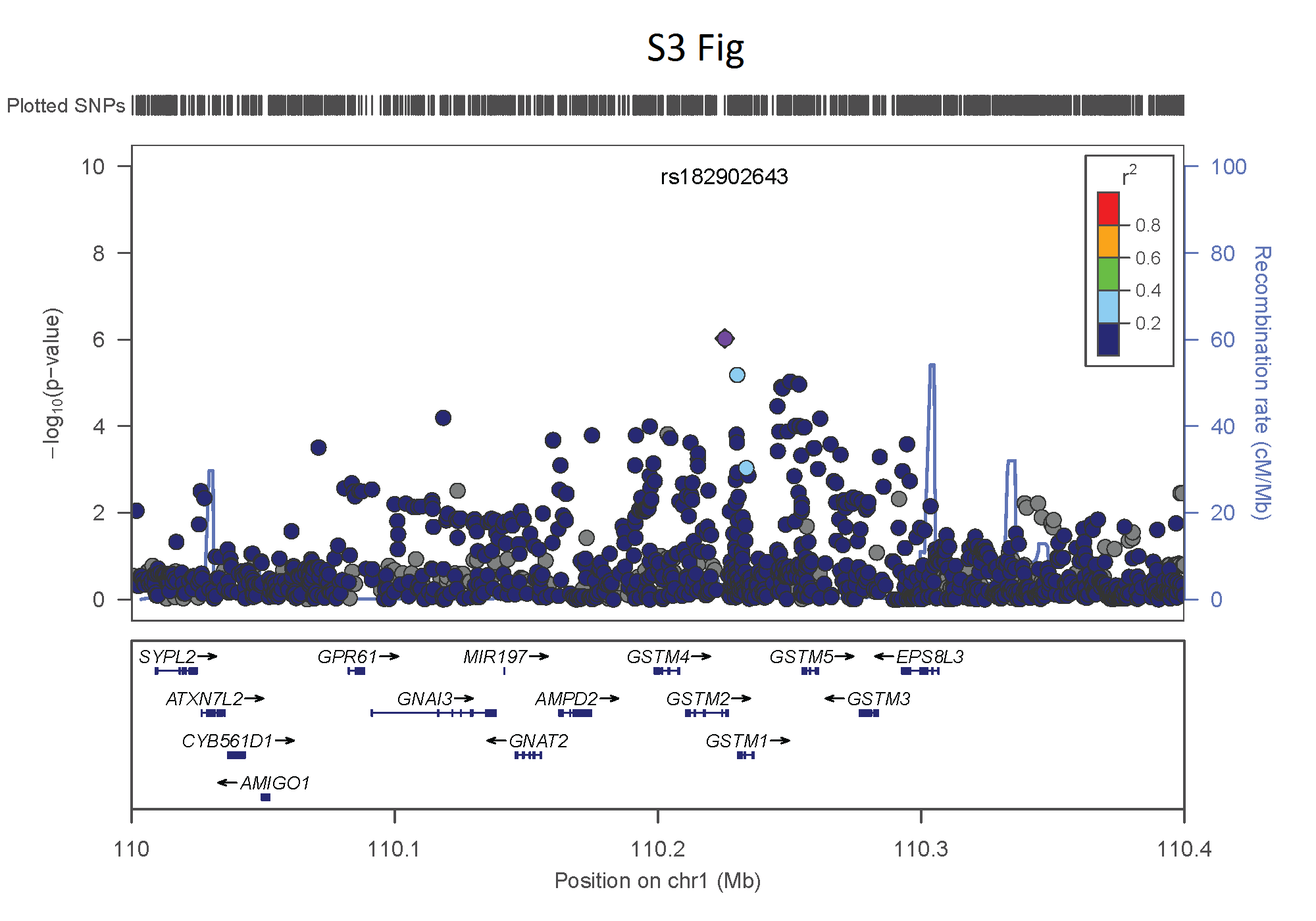

Supplement: S3 Fig — (TIF) [file pone.0150641.s003.tif]

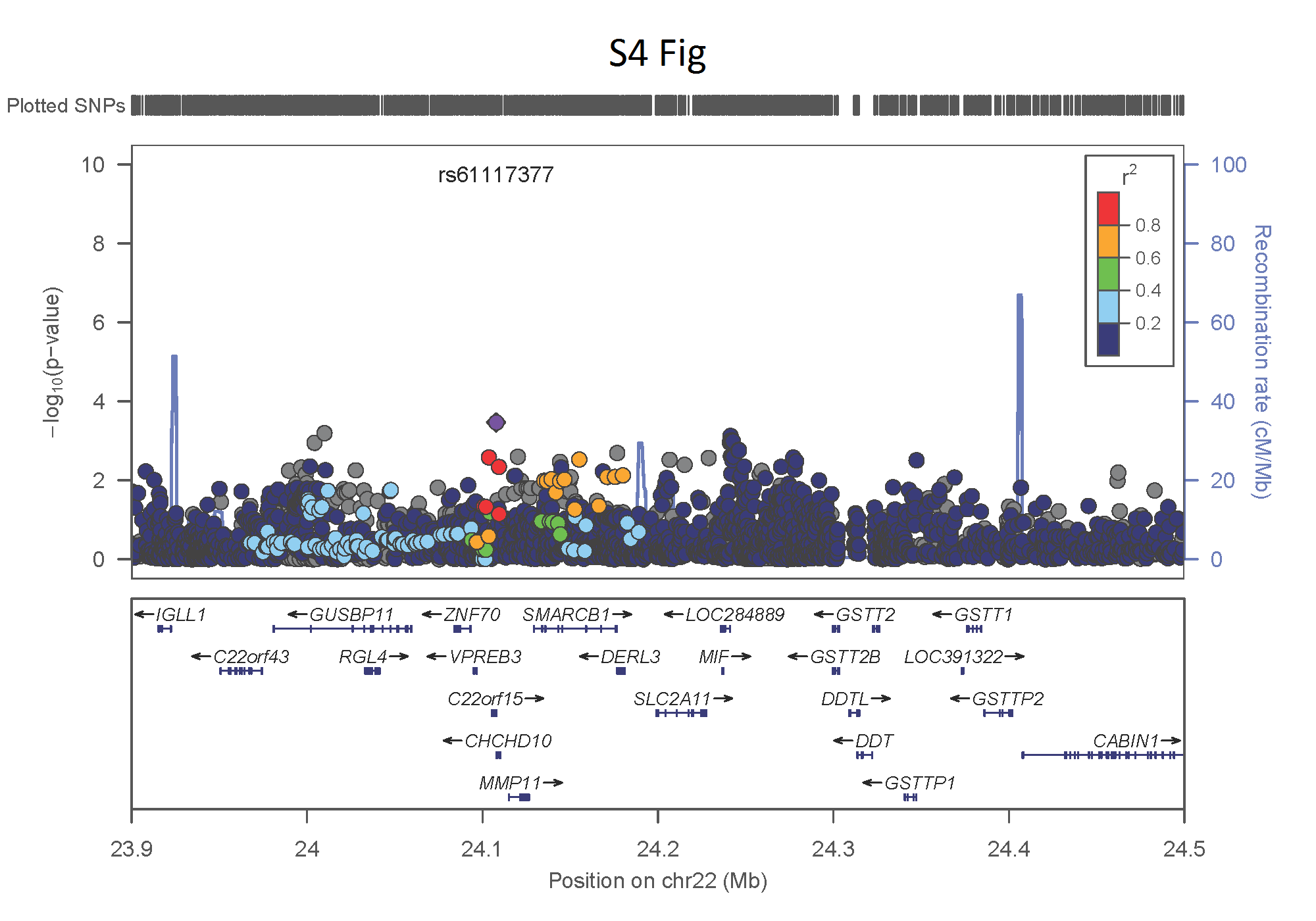

Supplement: S4 Fig — (TIF) [file pone.0150641.s004.tif]

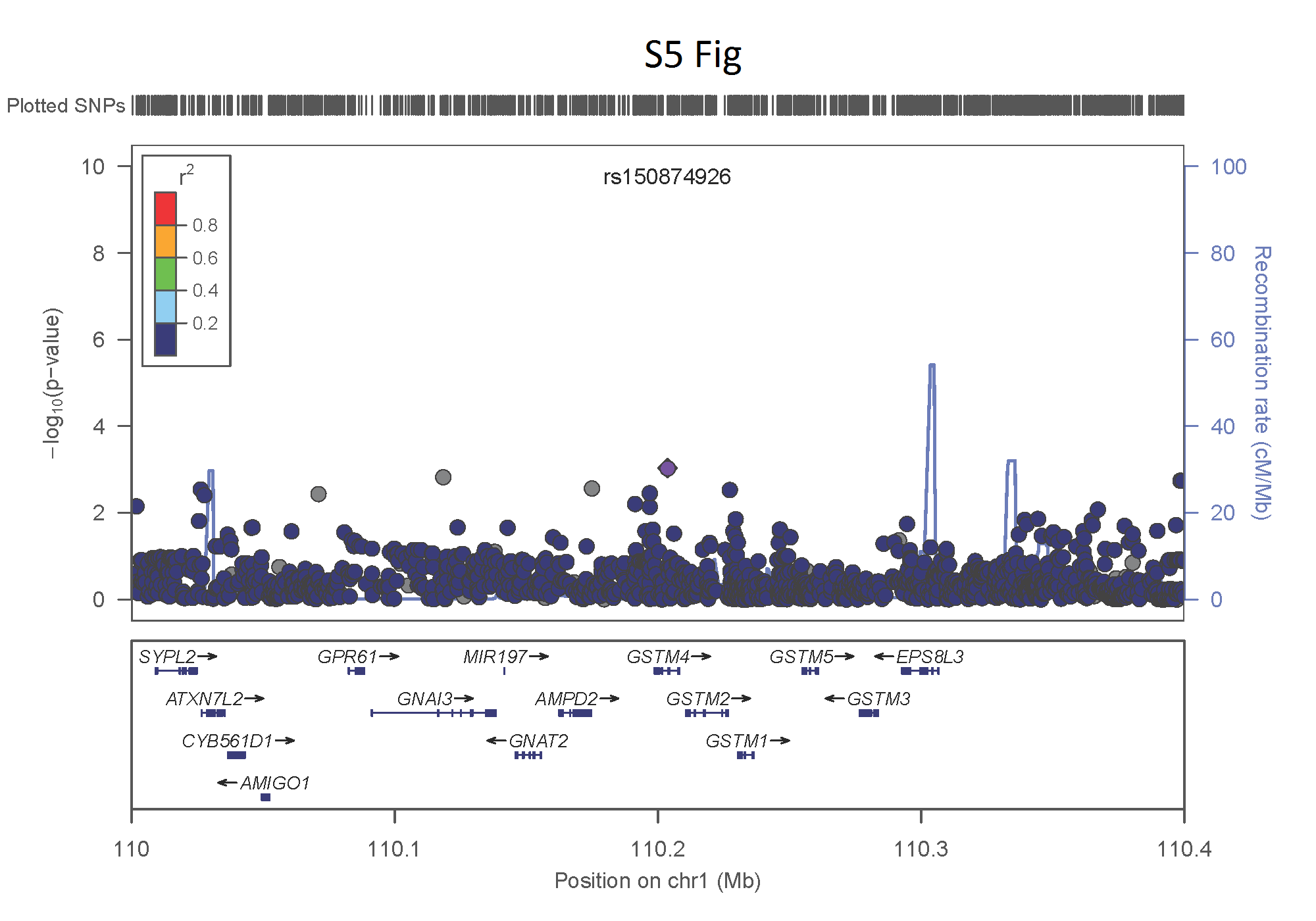

Supplement: S5 Fig — (TIF) [file pone.0150641.s005.tif]
